# Water Exchange Rate Shapes Intestinal Microbiota–Metabolite Interactions and Physiological Responses in *Babylonia areolata*

**DOI:** 10.3390/biology15171473

**Published:** 2026-09-01

**Authors:** Di Tang, Mingqiu Yang, Hongtao Liu, Feng Yu, Xin Zhan, Zhigang Tu, Minghui Shen

**Affiliations:** 1 Key Laboratory of Utilization and Conservation for Tropical Marine Bioresources of Ministry of Education, Hainan Tropical Ocean University, Sanya 572022, China; tangdi_hnhky@163.com (D.T.); ymq5338@163.com (M.Y.); xmulht@gmail.com (H.L.); 2 Hainan Provincial Key Laboratory of Tropical Maricultural Technologies, Hainan Academy of Ocean and Fisheries Sciences, Haikou 571126, China; 3 College of Life Sciences, Hainan Normal University, Haikou 571158, China; 4 School of Marine Biology and Fisheries, Hainan University, Haikou 570228, China; fengyu2022@hainanu.edu.cn (F.Y.); zhanxinuni@163.com (X.Z.)

**Keywords:** water exchange rate, *Babylonia areolata*, 16S rRNA gene sequencing, metabolomics, intestinal microbiome, physiological responses

## Abstract

In aquaculture, reduced water exchange helps constrain exogenous pathogen invasion, lower pumping costs, and improve management efficiency; nevertheless, its effects on the growth of cultured aquatic animals remain poorly characterized. This study compared two daily water-exchange rates (100% and 0%) to evaluate growth and associated intestinal responses in *Babylonia areolata* (ivory shell). Growth performance and intestinal enzyme activities were measured, while 16S rRNA gene sequencing and untargeted metabolomics were used to characterize microbial composition and metabolite profiles. The low-water-exchange group exhibited significantly lower weight gain rate (WGR, *p* < 0.0001), specific growth rate (SGR, *p* < 0.0001), daily increments in shell length (DISL, *p* = 0.0060), and shell width (DISW, *p* = 0.0012). Intestinal total superoxide dismutase (T-SOD, *p* < 0.0001) and lipase (LIP, *p* = 0.0011) activities were also significantly lower. The relative abundances of *Vibrio*, *Halodesulfovibrio*, and *Fusibacter* were higher, whereas that of *Christensenellaceae_R-7_group* was lower. Lipid-related metabolic pathways showed the most pronounced intestinal differences. Intestinal levels of prostaglandins (PGs), leukotrienes (LTs), and sphingosine (Sph) were significantly higher under low-water-exchange conditions. The relative abundance of *Halodesulfovibrio* correlated positively with PGs and LTs but negatively with lysophospholipids (Lpc) and T-SOD activity. The relative abundance of *Christensenellaceae_R-7_group* correlated positively with LIP activity and was significantly associated with metabolites involved in primary bile acid biosynthesis. Overall, low-water-exchange conditions were associated with coordinated changes in growth, intestinal enzyme activities, microbial composition, and metabolite profiles, although the underlying mechanisms require functional validation. These findings provide new insights into intestinal microbiota–metabolite responses to low-water-exchange conditions and may guide future validation and intervention studies in RAS-based ivory shell culture.

## Simple Summary

Reducing water exchange can limit the introduction of external pathogens and lower operating costs in aquaculture; however, its biological impacts on the ivory shell (*Babylonia areolata*) remain incompletely understood. In this experiment, we compared ivory shells cultured under 100% and 0% daily water exchange. Growth and intestinal enzyme activities were measured, while 16S rRNA gene sequencing and untargeted metabolomics were used to characterize microbial composition and metabolite profiles. Low water exchange was associated with poorer growth and lower intestinal total superoxide dismutase and lipase activities. In the low-water-exchange group, the relative abundances of *Vibrio*, *Halodesulfovibrio*, and *Fusibacter* were higher, whereas that of *Christensenellaceae_R-7_group* was lower. Lipid-related intestinal metabolites also differed, with higher levels of prostaglandins, leukotrienes, and sphingosine. *Halodesulfovibrio* abundance was positively associated with inflammation-related lipid metabolites but negatively associated with lysophospholipids and total superoxide dismutase activity. The relative abundance of *Christensenellaceae_R-7_group* was associated with bile-acid-related metabolites and positively correlated with lipase activity. Overall, low water exchange was associated with coordinated changes in growth, intestinal microbes, enzyme activities, and metabolites, although the underlying mechanisms require further investigation. These findings may inform future water-management strategies and research on intestinal microbiota in ivory shell culture.

## 1. Introduction

*Babylonia areolata* (ivory shell), a member of the family Babylonidae, is a warm-water gastropod widely distributed in the tropical and subtropical Indo-Pacific [1]. Because of its high economic value (approximately 100 yuan/kg) and fast growth, it is considered one of the most important aquaculture species and is widely farmed in China, Vietnam, and Thailand [2,3]. However, frequent disease outbreaks in ivory shell have recently posed major challenges to the industry [4,5]. Large-scale epidemics of diseases in aquaculture organisms are often linked to rapid aquaculture expansion, pollution of effluents, shared water intakes among farms, and farm-to-farm virus transmission [6]. Reducing water exchange rates is therefore regarded as an effective measure to limit exogenous viral invasion, improve management efficiency, and lower pumping costs [7].

Recirculating aquaculture systems (RASs) minimize water exchange to reduce external environmental impacts on aquaculture operations. Demonstration projects have been conducted for a range of species, including largemouth bass (*Micropterus salmoides*) [8], orange-spotted grouper (*Epinephelus coioides*) [9], olive flounder (*Paralichthys olivaceus*) [10], yellowtail kingfish (*Seriola lalandi*) [11], Atlantic salmon (*Salmo salar*) [12], and whiteleg shrimp (*Litopenaeus vannamei*) [13], and they have achieved favorable production benefits. Growth rate is a critical factor for the economic performance of commercial RASs. For shellfish, studies report significant differences (*p* < 0.05) in final size and survival of red abalone (Haliotis rufescens) between zero-water exchange RAS and flow-through aquaculture systems (FTS), with the growth rate of red abalone in the zero-water exchange RAS (26.1 μm/d) being significantly higher than that in the FTS (22.21 μm/d) [7]. Moreover, eastern oysters (*Crassostrea virginica*) was shown to grow stably in a zero-water exchange RAS, with survival exceeding 99% and weekly increases in length, width, and thickness of 1.3, 1.1, and 0.33 mm, respectively [14]. For fish, a study of European pikeperch (*Sander lucioperca*) in an RAS reported no significant differences in growth rate across three water exchange rates [6]. By contrast, Nile tilapia (*Oreochromis niloticus*) and european seabass (*Dicentrarchus labrax*) cultured in a zero-water exchange RAS experienced growth retardation. It is therefore suggested that water exchange rate substantially influences growth by altering the accumulation of organic and inorganic substances in high-density culture environments [15,16].

To date, studies of the culture environment for ivory shell have mainly concentrated on one or a few key environmental factors—temperature, salinity, pH, and ammonia nitrogen. Short-term single-factor or multi-factor orthogonal gradient stress experiments have analyzed the physiological and biochemical responses of ivory shells to acute mariculture stress [17,18]. However, no research has addressed the water exchange rate in RAS using medium-term mariculture experiments. In recent years, supported by local governments in China, the ivory shell industry has begun technological transformation. Increasingly, newly built modern fishery industrial parks (MFIPs) have adopted RASs with biological treatment for ivory shell farming to reduce water-pumping costs and prevent introduction of external pathogens. To evaluate the feasibility of cultivating ivory shell in RASs and assess growth differences between water-exchange regimes, we conducted a 60-day medium-term farming experiment. This duration is comparable to prior ivory-shell experiments in which significant growth and physiological responses were detected after seven to eight weeks [3,19]. We used 16S rRNA gene high-throughput sequencing and non-targeted liquid chromatography–mass spectrometry (LC–MS) metabolomics to characterize differences in intestinal microbial structure and metabolic function of ivory shell under different water exchange rates and to describe the organism’s associated responses under the zero-water-exchange RAS. This work provides preliminary applied practice and omics data support for RAS cultivation of ivory shell.

## 2. Materials and Methods

### 2.1. Ethics Statement

*Babylonia areolata* was obtained from the Qukou aquaculture breeding farm in Hainan Province, China. All research was conducted in accordance with the protocols of the Ethics Committee of the Hainan Academy of Ocean and Fisheries Sciences and was approved by the Laboratory Animal Ethics Committee of Hainan Academy of Ocean and Fisheries Sciences (EAEC-HAOFS-No.2026001; approval date: 9 May 2026).

### 2.2. Animal Housing and Experimental Design

The ivory shell used in the experiment originated from the Qukou Aquaculture Base in Haikou City, Hainan Province. Individuals had a mean body weight of 3.19 ± 0.16 g, a shell length of 23.32 ± 1.90 mm, and a shell width of 15.46 ± 1.54 mm. Before the aquaculture experiment, specimens were acclimated for 15 days at 27–28 °C, salinity 33–34, pH 7.9–8.1, and dissolved oxygen 7.05–7.28 mg/L. During acclimation, a commercial compound feed was provided daily at 3% of body weight. The proximate composition of the feed (Fuzhou Bohai Biotechnology Co., Ltd., Fuzhou, China) was 45.35 ± 0.28% crude protein, 10.10 ± 0.51% crude lipid, 8.17 ± 0.15% moisture, and 13.24 ± 0.24% ash. Water was completely exchanged each morning and evening, and dead snails, uneaten feed, and feces were removed promptly. Feeding was stopped 24 h before the experiment.

Based on local production experience and our preliminary experiments, a 100% daily water exchange rate is commonly used in traditional flow-through culture of ivory shell. Accordingly, two treatments were established: a high-exchange group (Group H, 100% daily water exchange) and a low-exchange group (Group L, zero daily water exchange). A small amount of freshwater was added to Group L only to maintain salinity. In this study, “zero daily water exchange” refers to the absence of scheduled water replacement rather than a completely closed system without any water input. Each treatment comprised six replicate plastic cages (20 × 15 × 24 cm), each serving as an experimental unit (*n* = 6). A total of 300 ivory shells were randomly allocated among the six cages in each treatment, with 50 individuals per cage.

### 2.3. Determination of Growth Parameters and Sample Collection

The experiment lasted for 60 days. Seventy-two hours after the final feeding, five ivory shells were randomly sampled from each cage (30 individuals per treatment), and their wet body weight, shell length, and shell width were measured individually. The mean value for each cage was calculated and used as the statistical unit for growth performance analyses (*n* = 6 per treatment). For tissue sampling, three individuals were randomly selected from each cage (18 individuals per treatment). After surface disinfection with 75% ethanol (Sinopharm Chemical Reagent Co., Ltd., Shanghai, China), the intestine, hepatopancreas, and muscle were aseptically collected. For each tissue type, samples from the three individuals within each cage were pooled to form one biological replicate and statistical unit, yielding six pooled samples per tissue per treatment. The pooled samples were immediately snap-frozen in liquid nitrogen (Hainan Jiateng Chemical Gas Co., Ltd., Haikou, China) and stored at −80 °C. All three tissue types were used for enzyme activity assays, and pooled intestinal samples were used for 16S rRNA gene sequencing and metabolomic analysis.

### 2.4. Growth Performance Calculations

The daily increment in shell length (DISL), daily increment in shell width (DISW), weight gain rate (WGR), and specific growth rate (SGR) were calculated as follows:

DISL(μm/day) = 1000 × [the final mean shell length (mm) − the initial mean shell length (mm)]/feeding days;

DISW(μm/day) = 1000 × [the final mean shell width (mm) − the initial mean shell width (mm)]/feeding days;

WGR(%) = 100 × [final body weight − initial body weight]/[initial body weight];

SGR(%/d) = 100 × [(ln final body weight − ln initial body weight)/feeding days].

### 2.5. Determination and Analysis of Enzyme Activity

Enzyme activities were measured in six biological replicates per tissue per treatment, with each replicate assayed in technical triplicate. For each sample, 0.5 g of tissue was homogenized in 1 mL of 0.5% (*w*/*v*) sodium chloride solution prepared with analytical-grade sodium chloride (Sinopharm Chemical Reagent Co., Ltd., Shanghai, China) using a Tissuelyser-48 L multi-sample tissue grinder (Shanghai Jingxin Industrial Development Co., Ltd., Shanghai, China). The homogenates were centrifuged at 4000 r/min for 10 min at 4 °C using a refrigerated Centrifuge 5425 R (Eppendorf AG, Hamburg, Germany). The supernatant was divided into two equal aliquots, placed in clean Eppendorf tubes to avoid repeated freeze–thaw cycles during measurement, and stored at −80 °C until analysis. Enzyme activities were measured using commercially available kits (Nanjing Jiancheng Bioengineering Institute, Nanjing, China) according to the manufacturer’s instructions: lipase (LIP, A054-2-1, microplate method), α-amylase (AMS, C016-2-1, microplate method), trypsin (TPS, A080-2-1, ultraviolet colorimetry), acid phosphatase (ACP, A060-2-1, microplate method), alkaline phosphatase (AKP, A059-2-1, microplate method), and total superoxide dismutase (T-SOD, A001-3-2, WST-1 method). Protein concentration was determined with the Coomassie Brilliant Blue Protein Assay Kit (A045-2-1). Absorbance was measured using a Synergy H1 multimode microplate reader (Agilent BioTek, Agilent Technologies, Inc., Santa Clara, CA, USA).

### 2.6. 16S rRNA Sequencing and Analysis of Gut Microbiota

Total DNA was extracted from intestinal samples using the FastPure Soil DNA Isolation Kit (magnetic bead) (MJYH, Shanghai, China) following the manufacturer’s instructions. The V3 and V4 regions of the 16S rRNA gene were amplified with primers 338F (ACTCCTACGGGAGGCAGCAG) and 806R (GGACTACHVGGGTWTCTAAT). PCR products were recovered on a 2% agarose gel and purified with a DNA gel recovery and purification kit (PCR Clean-Up Kit, Yuhua, Shanghai, China). The purified products were quantified using Qubit 4.0 (Thermo Fisher Scientific, Waltham, MA, USA). These PCR products were used for library construction with the NEXTFLEX Rapid DNA-Seq Kit, and sequencing was performed on the Illumina PE300/PE250 platform (Illumina, Inc., San Diego, CA, USA). Sequencing, quality control, DADA2 denoising, and annotation against the SILVA 16S rRNA gene database (v138) were carried out on the Majorbio Cloud Platform (Shanghai Majorbio Bio-Pharm Technology Co., Ltd., Shanghai, China) with a confidence threshold of 70%.

The Wilcoxon rank–sum test with false discovery rate (FDR) correction was used to compare differences in gut microbiota alpha diversity between groups, with adjusted *p* < 0.05 considered significant. Principal Coordinates Analysis (PCoA) based on Bray–Curtis distances was used to assess the similarity of microbial community structure among samples, and PERMANOVA was applied to test the significance of between-group differences. The linear discriminant analysis effect size (LEfSe) method was used to identify taxa with significantly different relative abundances between groups (http://huttenhower.sph.harvard.edu/LEfSe; accessed on 20 August 2026). A linear discriminant analysis (LDA) score > 4 and *p* < 0.05 were used as the significance thresholds. The Wilcoxon rank–sum test was used to identify bacterial genera with significantly different relative abundances between groups. Spearman correlation analysis of gut microbiota and gut enzyme activity indicators was performed using the R package vegan (v.2.5.3). Functional prediction of sequencing data was performed with PICRUSt2 (v.2.2.0) (https://github.com/picrust/picrust2/; accessed on 20 August 2026), and results were visualized using the R package vegan (v.2.5.3).

### 2.7. Non-Targeted Metabolomics and Analysis

Approximately 50 mg of intestinal tissue was placed into 2 mL centrifuge tubes. Then, 400 μL of extraction solution (methanol:water = 4:1 (*v*/*v*)) containing 0.02 mg/mL internal standard (L-2-chlorophenylalanine) was added. Samples were ground for 6 min under liquid nitrogen conditions (−10 °C, 50 Hz) and then subjected to low-temperature ultrasonic extraction for 30 min (5 °C, 40 kHz). The samples were incubated at −20 °C for 30 min and centrifuged for 15 min (4 °C, 13,000 g). The supernatant was transferred to sampling vials with inner cannulas for instrumental analysis. LC-MS analysis was performed on an ultra-high-performance liquid chromatography coupled with time-of-flight mass spectrometry (UPLC-TripleTOF) system from SCIEX (Framingham, MA, USA) at Shanghai Majorbio Bio-Pharm Technology Co., Ltd. (Shanghai, China). Chromatographic and mass spectrometric conditions were reported by Wang et al. [20]. Raw LC-MS data were imported into Progenesis QI (v.3.0; Waters Corporation, Milford, MA, USA) for preprocessing, and mass spectrometry (MS) data were matched to the public metabolite database Human Metabolome Database (HMDB) (http://www.hmdb.ca/; accessed on 20 August 2026) to obtain metabolite identities. Missing values in the data matrix were filtered out using the 80% rule. Response intensities of mass spectrometry peaks were normalized by the sum normalization method. Variables with a relative standard deviation (RSD) > 30% in quality control (QC) samples were deleted to improve metabolite identification and relative quantification.

Partial least squares–discriminant analysis (PLS-DA) was performed using the ropls package in R (v.1.6.2), and model stability was evaluated using seven-fold cross-validation. Metabolites with a variable importance in projection (VIP) score > 1 and *p* < 0.05 were considered differentially abundant metabolites (DAMs). Pathway enrichment analysis was conducted according to the Kyoto Encyclopedia of Genes and Genomes (KEGG) database (kegg_v20230830) (https://www.kegg.jp/kegg/pathway.html; accessed on 20 August 2026) using a Python package (v.1.0.0), and multiple testing correction was applied with the Benjamini–Hochberg (BH) method. Pathways with a nominal *p* value < 0.05 were considered nominally significant, whereas a BH-adjusted *p* value < 0.05 indicated enrichment that remained statistically significant after multiple-testing correction. KEGG pathway topology was analyzed using relative-betweenness centrality. Metabolomic results were visualized in R (v.4.1.2).

### 2.8. Data Analysis

Statistical analyses of growth and enzyme activity data were performed using SPSS Statistics 27.0. Data are presented as mean ± standard deviation (SD). Normality and homogeneity of variance were assessed using the Shapiro–Wilk and Levene’s tests, respectively. Differences between the H and L groups were evaluated using two-sided unpaired Student’s *t*-tests for normally distributed data with equal variances, Welch’s *t*-tests for normally distributed data with unequal variances, or Mann–Whitney U tests for non-normally distributed data. Differences were considered statistically significant at *p* < 0.05. Statistical methods and multiple-testing correction procedures for the microbiome and metabolomic datasets are described in Section 2.6 and Section 2.7, respectively.

## 3. Results

### 3.1. Effects of Water Exchange Rate on Growth Parameters and Enzyme Activities

After 60 days of culture, the final weight, final shell length, and final shell width in the H group were 5.12 ± 1.44 g, 27.63 ± 3.33 mm, and 18.88 ± 2.05 mm, respectively, which were significantly higher than those of the L group (4.55 ± 1.04 g, *p* < 0.0001; 26.67 ± 2.58 mm, *p* = 0.0060; 18.04 ± 1.78 mm, *p* = 0.0012). In the H group, SGR, WGR, DISL, and DISW were 1.10 ± 0.32%/day, 47.29 ± 9.75%, 101.32 ± 42.99 μm/d, and 74.11 ± 25.63 μm/d, respectively, all significantly higher than the values in the L group (0.62 ± 0.22%/day, *p* < 0.0001; 30.47 ± 9.54%, *p* < 0.0001; 70.96 ± 23.76 μm/d, *p* = 0.0060; 54.46 ± 17.69 μm/d, *p* = 0.0012), indicating better growth performance in the H group (Table 1). Endpoint pH and dissolved inorganic nitrogen concentrations (NO_3_^−^-N, NO_2_^−^-N, and NH_4_^+^-N) in the rearing water on day 60 are summarized in Table S1.

**Figure 1:**
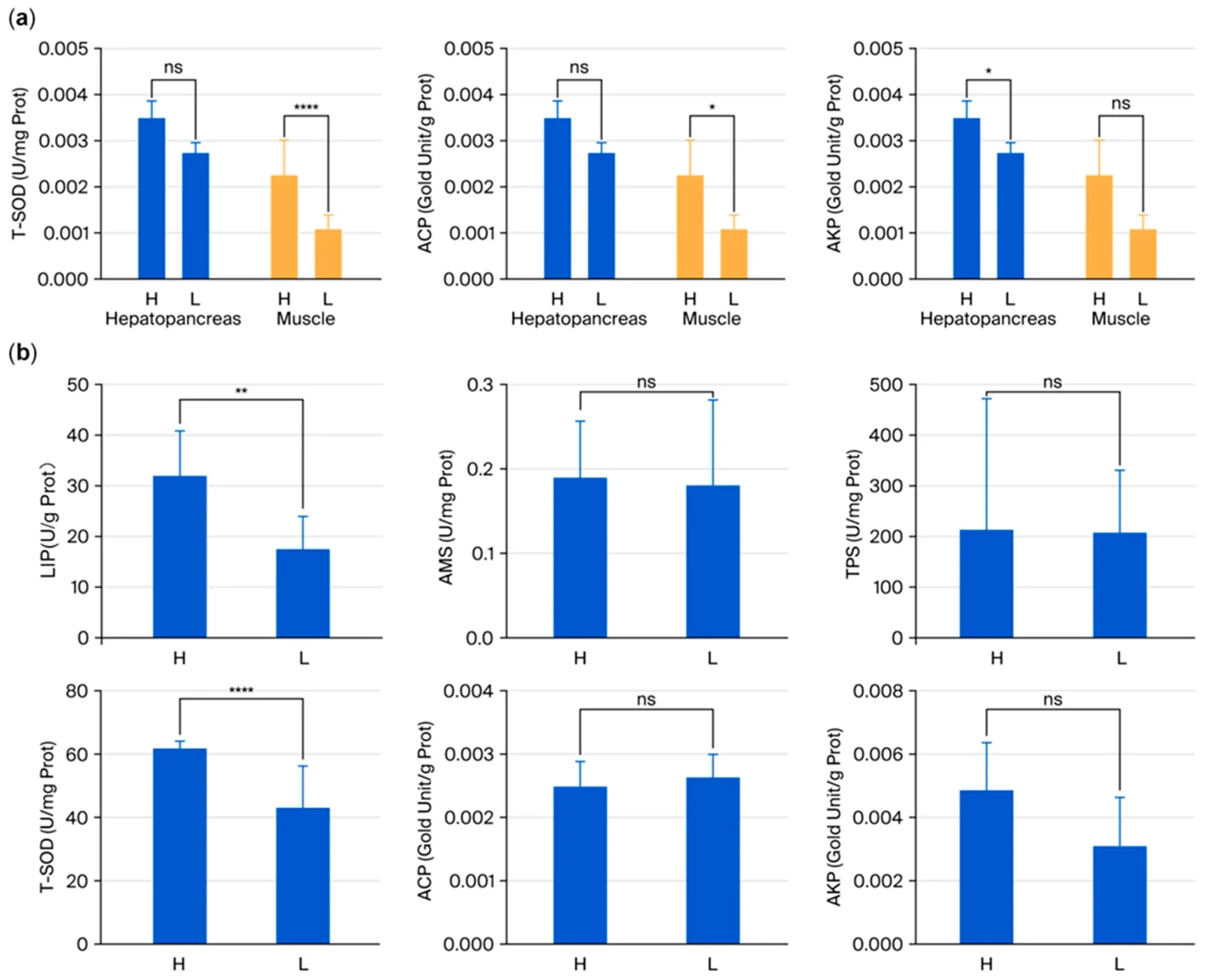
Enzyme activities of ivory shell. (**a**) Activities of T-SOD, ACP, and AKP in the hepatopancreas and muscle. (**b**) Activities of LIP, AMS, TPS, T-SOD, ACP, and AKP in the intestine; H and L indicate the high and low water exchange groups, respectively. LIP, lipase; AMS, α-amylase; TPS, trypsin; T-SOD, total superoxide dismutase; ACP, acid phosphatase; AKP, alkaline phosphatase. Data are presented as mean ± SD. ns, not significant; * *p* < 0.05, ** *p* < 0.01, **** *p* < 0.0001.

The activities of three digestive enzymes (LIP, AMS, and TPS) in intestinal tissue and those of three immune/antioxidant enzymes (ACP, AKP, and T-SOD) in intestinal, hepatopancreatic, and muscle tissues were compared between the H and L groups (Figure 1). In intestinal tissue, LIP and T-SOD activities in the L group were significantly lower than those in the H group (*p* = 0.0011 and *p* < 0.0001, respectively), whereas AMS, TPS, ACP, and AKP showed no significant differences (*p* > 0.05). In hepatopancreas and muscle tissues, only hepatopancreatic AKP and muscle T-SOD and ACP activities were significantly lower in the L group compared with the H group (*p* = 0.0231, *p* < 0.0001 and *p* = 0.0411, respectively); activities of the remaining enzymes did not differ significantly (*p* > 0.05).

### 3.2. Effects of Water Exchange Rate on Intestinal Microbiota

**Figure 2:**
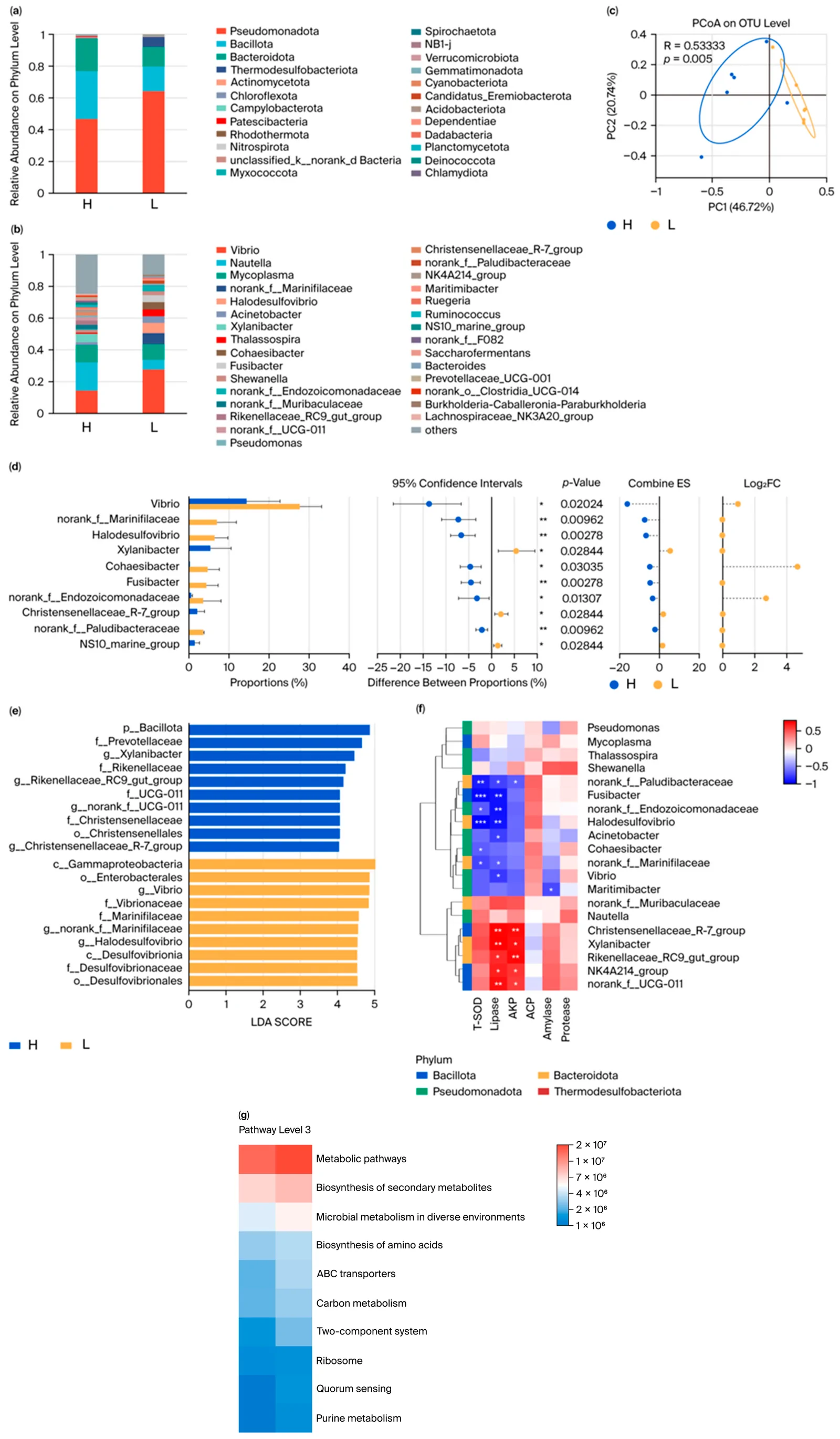
The structure and function of the intestinal microbiota in the H and L groups. (**a**) Relative abundance of intestinal microbiota at the phylum level; (**b**) relative abundance of intestinal microbiota at the genus level; (**c**) principal coordinates analysis (PCoA) plot demonstrating the overall compositional similarities of the intestinal microbiota; (**d**) Wilcoxon rank–sum test bar plot of differential taxa at the phylum and genus levels; (**e**) linear discriminant analysis effect size (LEfSe) analysis identifying significantly enriched microbial taxa; (**f**) correlation analysis of gut enzyme activity and intestinal microbes; (**g**) Phylogenetic Investigation of Communities by Reconstruction of Unobserved States 2 (PICRUSt2)-predicted Kyoto Encyclopedia of Genes and Genomes (KEGG) Level 3 functional profiles. The left and right columns represent the H and L groups, respectively. The color gradient represents the relative abundance of predicted functions, as indicated by the legend. H, high water-exchange group; L, low water-exchange group. * *p* < 0.05, ** *p* < 0.01, *** *p* < 0.001.

To assess differences in the intestinal microbiota of ivory shell between water-exchange regimes, high-throughput sequencing was performed on 12 intestinal samples from groups H and L, generating 428,088 valid sequences. After sequence filtering, taxonomic annotation, and microbial community clustering, the intestinal microbiota composition in groups H and L was dominated at the phylum level by Pseudomonadota (46.67%, 64.15%), Bacillota (30.13%, 15.61%), and Bacteroidota (20.54%, 12.23%), together accounting for over 90% of the community and representing the core intestinal microbiota of ivory shell (Figure 2a). At the genus level, the core microbiota of ivory shell differed between the two water exchange groups. The relative abundances were as follows: *Nautella* (17.70%, 6.12%), *Vibrio* (14.35%, 27.65%), *Mycoplasma* (10.95%, 9.83%), *Acinetobacter* (1.76%, 4.21%), *Thalassospira* (0.68%, 4.26%), *Cohaesibacter* (0.16%, 4.63%), *Shewanella* (1.73%, 2.54%), *norank_f__Endozoicomonadaceae* (0.53%, 3.50%), *norank_f__Muribaculaceae* (2.57%, 0.20%), *norank_f__UCG-011* (2.10%, 0.01%), *Pseudomonas* (1.08%, 1.00%), *NK4A214_group* (1.76%, 0.01%), *Maritimibacter* (0.60%, 1.14%), *Ruegeria* (0.90%, 0.67%), *Ruminococcus* (1.39%, 0.01%), *Bacteroides* (0.20%, 0.84%), *Prevotellaceae_UCG-001* (0.99%, 0.01%), *norank_o__Clostridia_UCG-014* (0.94%, 0.06%), and *Burkholderia-Caballeronia-Paraburkholderia* (0.09%, 0.90%), which together accounted for more than 70% of the total microbial community (Figure 2b).

Alpha diversity indices reflect species richness and diversity within microbial communities. There were no significant differences in the Chao, Sobs, ACE, Shannon, and Simpson indices of intestinal samples between the H group and the L group (FDR-adjusted *p* > 0.05), as shown in Table 2. These findings indicate that the two water-exchange groups did not differ significantly in intestinal microbial richness or diversity. Beta diversity analysis based on Bray–Curtis distances showed that intestinal samples from the H and L groups formed distinct clusters and that their community structures differed significantly (*p* = 0.05; Figure 2c), indicating significant compositional differences between the two water-exchange groups. Differences in intestinal bacterial genera of ivory shell between the two water exchange rates were further examined using the Wilcoxon rank–sum test and LEfSe multilevel species discriminant analysis. Differential community analysis (Figure 2d) showed that the L group had significantly higher intestinal relative abundances of *Vibrio*, *norank_f__Muribaculaceae*, *Halodesulfovibrio*, *Cohaesibacter*, *Fusibacter*, *norank_f__Endozoicomonadaceae*, *and norank_f__Paludibacteraceae*. By contrast, the H group exhibited significantly higher intestinal relative abundances of *Xylanibacter*, *Christensenellaceae_R-7_group*, and *NS10_marine_group* compared with the L group. LEfSe analysis (Figure 2e) corroborated these findings, identifying *Xylanibacter*, *Rikenellaceae_RC9_gut_group*, *g__norank_f__UCG-011*, and *Christensenellaceae_R-7_group* as marker taxa for the high exchange condition, and *Vibrio*, *norank_f__Marinifilaceae*, and *Halodesulfovibrio* as marker taxa for the low exchange condition.

To examine associations between gut microbial communities and host physiological measures under different water exchange rates, the Bray–Curtis community distance metric and the Euclidean environmental factor metric were used to assess correlations between intestinal microbes and digestive enzymes at the genus level. The results (Figure 2f) showed that LIP and AKP were predominantly and significantly positively correlated with genera enriched under the high-exchange condition, including *g__norank_f__UCG-011*, *NK4A214_group*, *Rikenellaceae_RC9_gut_group*, *Xylanibacter*, and *Christensenellaceae_R-7_group*. T-SOD and LIP were significantly negatively correlated with *Vibrio*, *norank_f__Marinifilaceae*, *Cohaesibacter*, *norank_f__Endozoicomonadaceae*, *Halodesulfovibrio*, *Fusibacter*, *norank_f__Paludibacteraceae*, and others. PICRUSt2 was used to infer the functional potential of the intestinal microbiota from 16S rRNA gene sequencing data (Figure 2g). In both water-exchange groups, the predominant predicted functions included metabolic pathways, biosynthesis of secondary metabolites, microbial metabolism in diverse environments, and biosynthesis of amino acids. These predictions represent putative functional potential rather than direct measurements of gene expression or metabolic activity.

### 3.3. Effects of Water Exchange Rate on Intestinal Metabolomics

**Figure 3:**
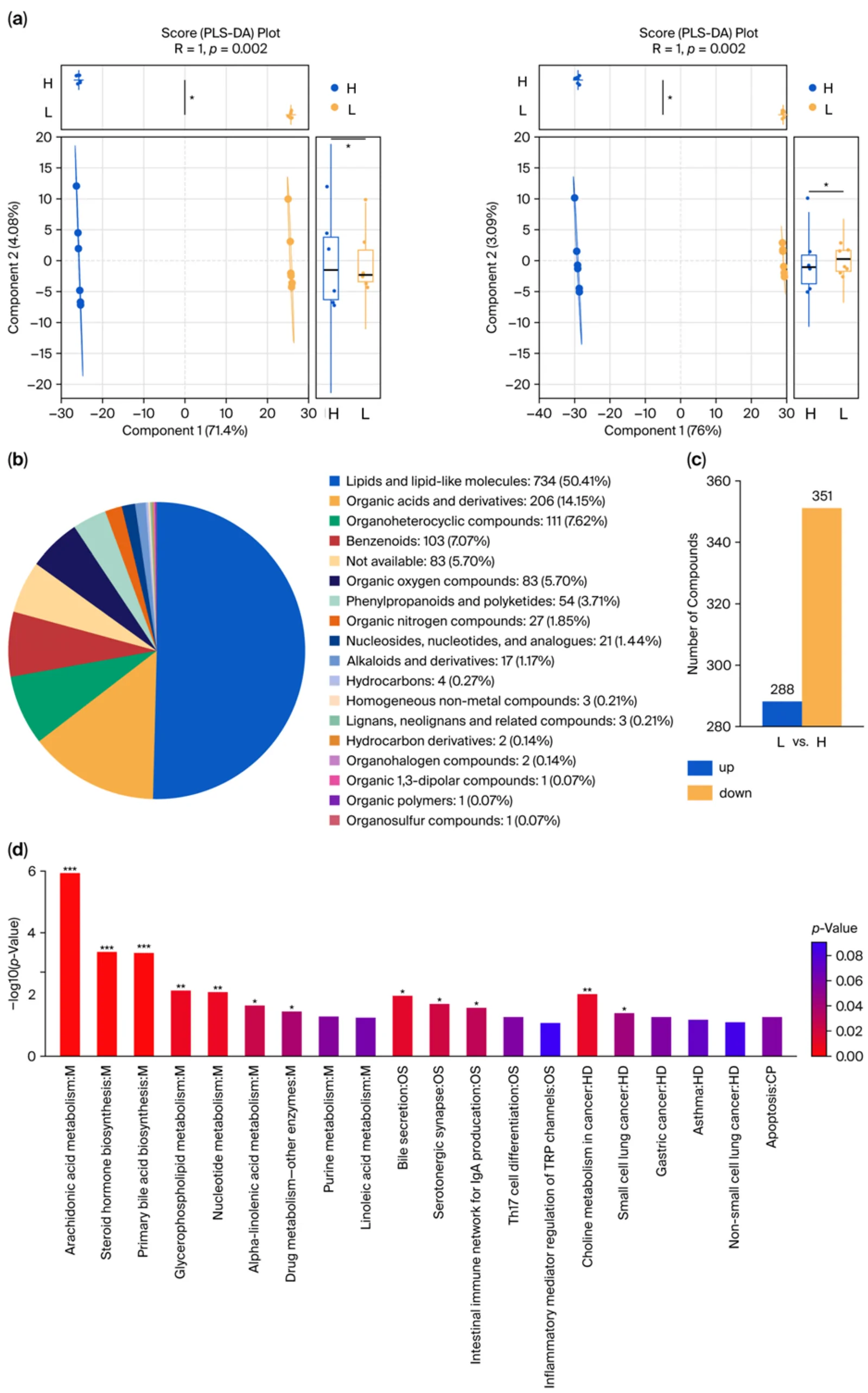

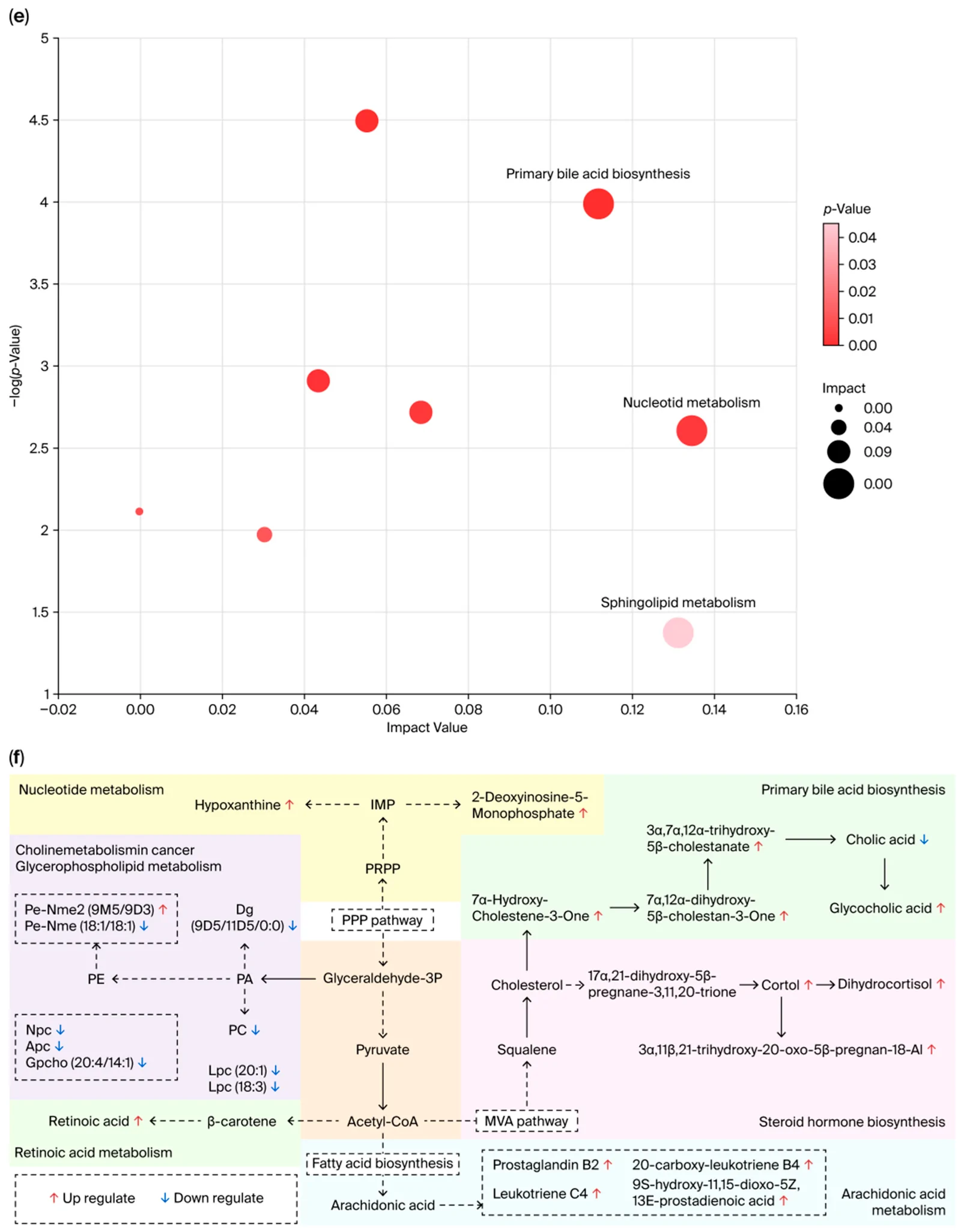

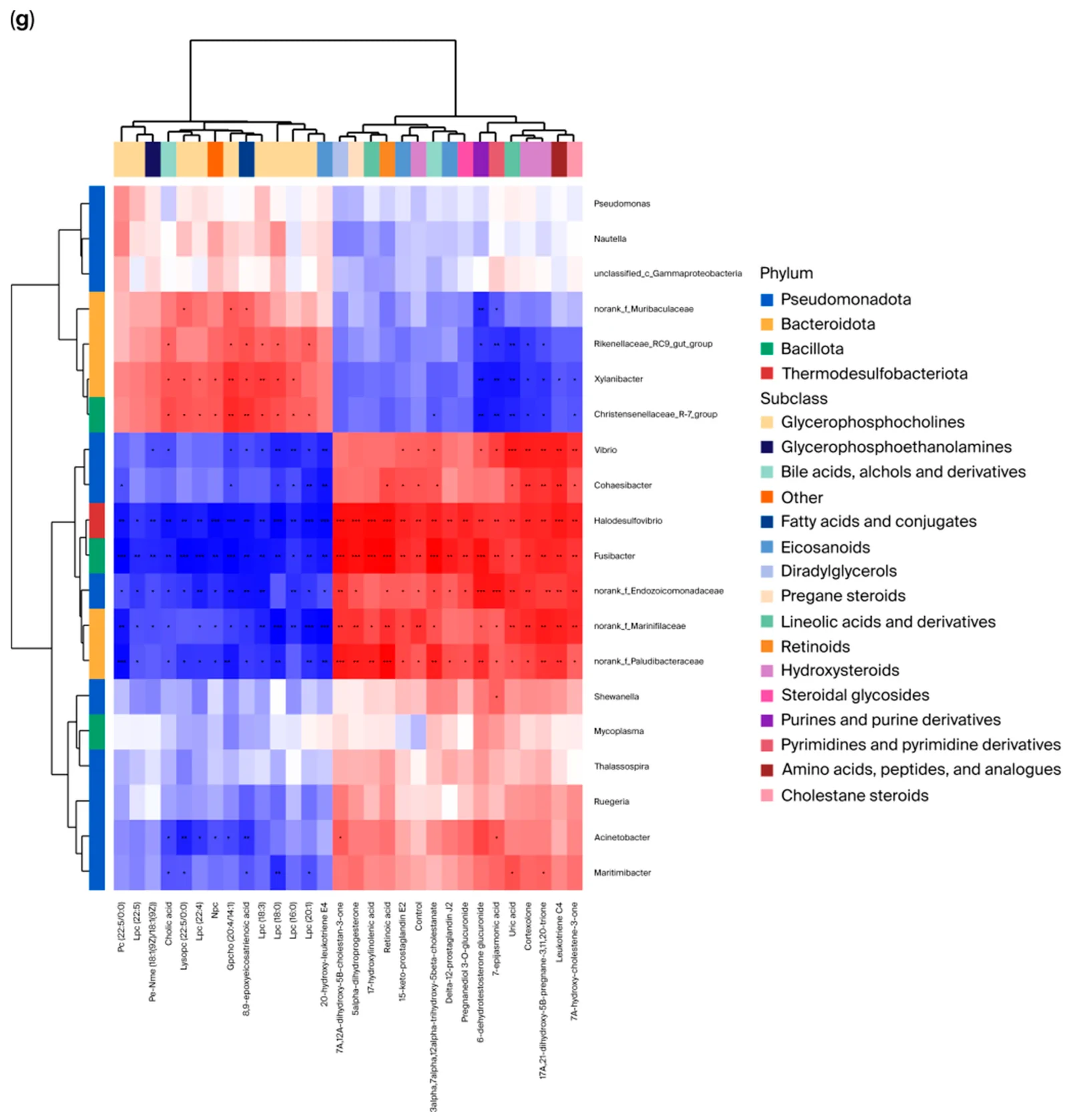
Metabolomic analysis of intestinal tissues in the H and L groups. (**a**) Partial least squares discriminant analysis (PLS-DA) score plot for positive ions (**left**) and negative ions (**right**); the marginal boxplots above and to the right show the distributions of sample scores along the corresponding PLS-DA components, the points represent individual samples, and the black horizontal line within each box indicates the median. (**b**) Human Metabolome Database (HMDB) classification of differentially abundant metabolites; (**c**) stacked bar chart of differentially abundant metabolites (DAMs) in the H and L groups; (**d**) Kyoto Encyclopedia of Genes and Genomes (KEGG) pathway enrichment analysis of DAMs; (**e**) relative betweenness centrality of KEGG functional pathway in the H and L groups; (**f**) network analysis of intestinal metabolic pathways affected by water exchange rate in ivory shell, with solid arrows indicating direct metabolic conversions and dashed arrows indicating multistep conversions involving intermediate metabolites; (**g**) correlation analysis of gut intestinal microbes and DAMs in the H and L groups. DAMs were identified using a variable importance in projection (VIP) score ≥ 1, fold change (FC ≥ 1 or FC ≤ 1), and *p* < 0.05. H, high water-exchange group; L, low water-exchange group. * *p* < 0.05, ** *p* < 0.01, *** *p* < 0.001.

Intestinal metabolomic profiling of the H and L groups identified a total of 2250 known metabolites. PLS-DA results show a clear separation between the H and L groups (Figure 3a), and analysis of similarities (ANOSIM) confirmed a significant difference in intestinal metabolite profiles between the groups (R = 1, *p* = 0.002). HMDB classification of the differential metabolites showed that lipids and lipid-like molecules (50.41%), organic acids and their derivatives (14.15%), and organic heterocyclic compounds (7.62%) were the three largest categories among the differential abundant metabolites (DAMs) (Figure 3b). To identify intestinal metabolic pathways associated with water exchange rate, metabolites and pathways that differed significantly between the two groups were compared using PLS-DA (VIP ≥ 1), univariate fold change (FC ≥ 1 or FC ≤ 1), and statistical significance (*p* < 0.05). Wilcoxon rank–sum test (*p* < 0.05) indicated that, compared to the H group, the L group showed significant upregulation of 288 metabolites and significant downregulation of 351 metabolites (Figure 3c). These differential metabolites were mapped to KEGG pathways for enrichment analysis to identify biological processes associated with growth and metabolism under different water-exchange conditions. KEGG pathway enrichment analysis identified 12 pathways with nominal significance (nominal *p* value < 0.05; Figure 3d), including five lipid metabolism pathways: arachidonic acid metabolism, steroid hormone biosynthesis, primary bile acid biosynthesis, glycerophospholipid metabolism, and alpha-linolenic acid metabolism. The remaining pathways were nucleotide metabolism, choline metabolism in cancer, bile secretion, serotonergic synapse, intestinal immune network for IgA production, drug metabolism–other enzymes, and small cell lung cancer (Table 3). Of these, arachidonic acid metabolism, steroid hormone biosynthesis, and primary bile acid biosynthesis remained statistically significant after BH correction (BH-adjusted *p* value < 0.05), whereas the other nine pathways were retained as exploratory findings based on nominal significance. Topological analysis based on relative betweenness centrality (Figure 3e) indicated that three differential metabolic pathways—primary bile acid biosynthesis, nucleotide metabolism, and sphingolipid metabolism—had relatively high impact values (0.112, 0.134, and 0.131, respectively). These pathways were therefore highlighted as having relatively high topological importance in the intestinal metabolic network.

A network diagram of intestinal metabolic pathways differing between water-exchange groups was constructed (Figure 3f). Under low-water-exchange conditions, metabolites involved in arachidonic acid metabolism—20-Carboxy-Leukotriene B4, Prostaglandin B2, and 9S-Hydroxy-11,15-Dioxo-5Z,13E-Prostadienoic Acid—were significantly upregulated, while those involved in choline metabolism in cancer and in glycerophospholipid metabolism—Npc, Apc, Lpc (20:1), and Lpc (18:3)—were significantly downregulated. In primary bile acid biosynthesis, 3Alpha,7Alpha,12Alpha-Trihydroxy-5Beta-Cholestanate and Glycocholic Acid were significantly upregulated, whereas Cholic Acid was significantly downregulated. Cortol, Dihydrocortisol, and 3A,11B,21-Trihydroxy-20-Oxo-5B-Pregnan-18-Al in steroid hormone biosynthesis were also significantly upregulated. Finally, 2′-Deoxyinosine 5′-Monophosphate in nucleotide metabolism was significantly upregulated. These coordinated metabolite changes were associated with low-water-exchange conditions and may reflect alterations in intestinal lipid metabolism, inflammation-related processes, and nucleotide metabolism, although their physiological significance requires further functional validation.

To characterize associations between differentially abundant gut bacterial genera and intestinal DAMs across the two water-exchange conditions, we performed a correlation analysis (Figure 3g). Based on their correlations with the DAMs, the bacterial genera formed two distinct clusters. One cluster was dominated by marker genera associated with a high water exchange rate, including *Xylanibacter*, *Rikenellaceae_RC9_gut_group*, *g__norank_f__UCG-011*, and *Christensenellaceae_R-7_group*. The other cluster was composed primarily of marker genera associated with a low water exchange rate, such as *Vibrio*, *norank_f__Marinifilaceae*, and *Halodesulfovibrio*. Gut metabolites were also separated into two main groups. One group consisted mainly of metabolites that were significantly downregulated in the Choline metabolism in cancer and Glycerophospholipid metabolism pathways, for example Lpc, NPC, and Gpcho (20:4/14:1). The other group was dominated by metabolites that were significantly upregulated in the Primary bile acid biosynthesis, Arachidonic acid metabolism, and Steroid hormone biosynthesis pathways, such as 3Alpha,7Alpha,12Alpha-Trihydroxy-5Beta-Cholestanate, Leukotriene C4, and 3A,11B,21-Trihydroxy-20-Oxo-5B-Pregnan-18-Al. As shown in Figure 3g, metabolites that were significantly downregulated correlated positively with gut marker microorganisms at high water exchange rates and negatively at low water exchange rates, whereas significantly upregulated metabolites displayed the opposite pattern. Collectively, these findings indicate coordinated associations between gut microbial taxa and intestinal metabolite profiles under different water-exchange conditions, without establishing causal or directional relationships.

## 4. Discussion

Aquaculture conditions can shape the composition, structure, and function of the intestinal microbiota, with potential consequences for host nutrient acquisition, energy metabolism, and immune function [21]. Previous studies have examined the culture requirements of ivory shell and its responses to temperature, salinity, pH, and ammonia nitrogen [17,18,19]. However, the effects of water-exchange regimes on its intestinal microbiota and metabolite profiles remain poorly understood. In the present study, the low-water-exchange group exhibited significantly lower SGR, WGR, DISL, and DISW than the high-water-exchange group. A comparable reduction in growth was reported in European sea bass reared in recirculating rather than flow-through systems [15]. Poorer growth under low-water-exchange conditions coincided with shifts in intestinal microbial composition and metabolite profiles, together with lower intestinal LIP and T-SOD activities. These coordinated responses suggest that poorer growth may be associated with a reduced capacity for intestinal lipid digestion and antioxidant defense, alongside microbial and metabolic alterations.

The intestinal microbiota is essential for host physiological functions, including nutrient acquisition, development, homeostasis, and immunity [22]. At the phylum level, the core intestinal microbiota of ivory shell under two water exchange rates remained Pseudomonadota, Bacillota, and Bacteroidota, consistent with previous findings in ivory shell and other aquaculture organisms [23,24,25]. Previous studies have associated Bacillota and Bacteroidota with lipid and carbohydrate metabolism, respectively [26,27]. The Bacillota/Bacteroidota ratio has been proposed as a potential indicator of nutrient absorption efficiency in cultured animals [28]. In this study, this ratio was lower in the low-water-exchange group than in the high-water-exchange group (1.23 vs. 1.47), and intestinal LIP activity was also significantly lower in the low-water-exchange group (*p* = 0.0011). These changes coincided with poorer growth under low-water-exchange conditions; however, whether the observed differences in the gut microbiota are associated with changes in LIP activity, nutrient absorption, or growth remains to be determined.

At the genus level, the relative abundances of potentially pathogenic genera, including *Vibrio* and *Halodesulfovibrio*, were significantly higher in the intestines of the low-water-exchange group than in those of the high-water-exchange group. The genus *Vibrio* includes important opportunistic pathogens in aquaculture that can express virulence factors and infect invertebrates [29,30]. Members of *Halodesulfovibrio* are sulfate-reducing bacteria (SRB) that use sulfate as an electron acceptor and produce hydrogen sulfide (H_2_S) during metabolism. Evidence from other biological models suggests that H_2_S exposure is associated with DNA damage and alterations in inflammatory, oncogenic, proliferative, and apoptotic signaling [31,32]. By contrast, *Xylanibacter*, *Rikenellaceae_RC9_gut_group*, *g__norank_f__UCG-011*, and *Christensenellaceae_R-7_group* were significantly more abundant in the intestines of the high-water-exchange group. *Xylanibacter* has been reported as a dominant member of the rumen microbiota capable of degrading hemicellulose, pectin, and energy-storage polysaccharides in plant cell walls [33]. *Rikenellaceae_RC9_gut_group* has been associated with variations in intestinal acetate levels, and its relative abundance may be influenced by dietary composition and environmental conditions [34,35,36]. *Christensenellaceae*, represented by *Christensenella minuta*, is among five bacterial groups proposed as indicators of a healthy gut microbiome [37]. Under anaerobic conditions, cultured *C. minuta* can ferment glucose into short-chain fatty acids, including acetate and butyrate [38]. It has also been associated with favorable metabolic characteristics, including lower body mass index, serum lipid levels, and visceral adiposity. Although these microbial taxa have been associated with potentially pathogenic or probiotic properties in other host systems, their effects on digestive capacity, intestinal health, or growth in ivory shell require experimental validation.

The intestine plays important roles in digestion, nutrient absorption, barrier function, and immune regulation [22]. Interactions among the intestinal microbiota, microbial metabolites, and the mucosal immune system contribute to intestinal immune homeostasis and barrier integrity [39]. In the low-water-exchange group, genera containing potentially pathogenic members were significantly more abundant. Among the arachidonic acid metabolites, Prostaglandin B2 and Leukotriene C4 were significantly more abundant, and showed significant positive correlations with the relative abundances of *Vibrio* and *Halodesulfovibrio* (Figure 3g). Prostaglandins (PGs) and leukotrienes (LTs) are n-6 PUFA-derived eicosanoids closely associated with inflammatory signaling in other biological systems [40]. However, intestinal inflammation was not directly assessed in this study. Therefore, the observed changes in PGs and LTs and their correlations with bacterial taxa indicate only associations with inflammation-related metabolic processes and do not demonstrate intestinal inflammation in ivory shell. Direct experimental assessment is required to determine the presence or absence of intestinal inflammation under low-water-exchange conditions. In addition, phosphatidylcholine (PC) and its derivative lysophospholipids (Lpc), assigned to glycerophospholipid metabolism and choline metabolism in cancer, were downregulated in the low-water-exchange group. Lpc is produced from PC by phospholipase A2 and is a major lipid component of oxidized low-density lipoprotein (LDL) [41]. Studies in other biological models suggest that microbiota-derived Lpc may enhance Nrf2-related antioxidant responses involving SOD and CAT, potentially alleviating oxidative stress and apoptosis [42,43,44]. In this study, several Lpc species and intestinal T-SOD activity were significantly lower in the low-water-exchange group and showed significant negative correlations with the relative abundances of *Halodesulfovibrio* and *Fusibacter*. These findings indicate associations between the measured Lpc and T-SOD changes and the relative abundances of these genera. Whether these genera contribute functionally to these changes or to intestinal immune or barrier responses requires further validation. It should be noted that disease-related KEGG pathway labels, including choline metabolism in cancer and small cell lung cancer, denote conserved molecular networks rather than corresponding pathological processes in mollusks. Their biological significance should therefore be interpreted cautiously, with emphasis on the underlying metabolites and conserved biochemical processes.

Nucleotide metabolism was nominally significant under low-water-exchange conditions (nominal *p* value = 0.009, BH-adjusted *p* value = 0.128) and exhibited a relatively high impact value of 0.134. Purine metabolites, including hypoxanthine and 2′-deoxyinosine 5′-monophosphate (dIMP), were more abundant in the low water exchange group. Purines serve central roles in cellular energy metabolism and signal transduction and can function as signaling molecules that activate immune cells and induce the release of inflammatory mediators during inflammation [45,46]. Although intestinal microbial purine metabolism has been linked to host metabolic and inflammatory status in other systems [47], this study did not directly measure microbial purine-metabolic activity or assess intestinal inflammation. Therefore, the present data do not establish that microbial purine metabolism contributed to the observed purine changes or that these changes indicate intestinal inflammation in ivory shell. Pathway topology analysis also highlighted sphingolipid metabolism (nominal *p* value = 0.0188, impact value = 0.131). Sphingosine (Sph), a fundamental component of sphingolipids, was more abundant in the intestines of the low-water-exchange group. Previous studies suggest that Sph contributes to membrane lipid composition and may mediate host–microbe interactions [48]. Its increased abundance suggests an association between sphingolipid metabolism and the intestinal response to low water exchange. Previous studies have implicated Sph in apoptotic signaling [49]. However, whether increased Sph abundance is associated with intestinal apoptotic pathway activation in ivory shell remains to be determined.

Primary bile acid biosynthesis was one of the three pathways with relatively high topological importance and one of the three that remained statistically significant after BH correction (nominal *p* value = 0.0005; BH-adjusted *p* value = 0.01575; impact value = 0.112). Bile acids (BAs) can activate pancreatic lipase in the intestine, bind to triglycerides (TG) surfaces, and facilitate lipid digestion and utilization [50]. A previous study reported that *Christensenella minuta* can acylate host-derived bile acids, including cholic acid (CA) and deoxycholic acid (DCA), in vitro [51]. In that study, the resulting 3-O-acylated bile acids inhibited intestinal FXR signaling, and administration of *C. minuta* alleviated features of metabolic disease in high-fat-diet-induced obese mice [51]. In the present study, the relative abundance of *Christensenellaceae_R-7_group* was significantly correlated with several metabolites involved in primary bile acid biosynthesis. These metabolites included Cholic Acid, 7A-Hydroxy-Cholestene-3-One, 7A,12A-Dihydroxy-5B-Cholestan-3-One, and 3Alpha,7Alpha,12Alpha-Trihydroxy-5Beta-Cholestanate. The relative abundance of *Christensenellaceae_R-7_group* abundance was also significantly and positively correlated with intestinal LIP activity (Figure 2f). These findings indicate that *Christensenellaceae_R-7_group* abundance was associated with LIP activity and bile-acid-related metabolites in ivory shell. However, direct experimental validation is required to determine the specific functions of this microbial taxon in the intestine of ivory shell and whether it contributes to the changes in intestinal enzyme activities and metabolite profiles measured in this study.

In summary, this study integrated microbiome and metabolome analyses to characterize differences in the intestinal microbial community and metabolic pathways of ivory shell under low-water-exchange conditions. These findings provide new insights into intestinal microbiota–metabolite associations and may inform the optimization of RAS-based ivory shell culture. Taxa enriched under high-water-exchange conditions may serve as sources of candidate strains for future functional evaluation. Following experimental validation of their probiotic efficacy, safety, and ability to mitigate the effects of low water exchange, selected strains may be considered for use as feed additives. In addition, the water-exchange-associated metabolic pathways identified here may inform future mechanistic and intervention studies in RAS-cultured gastropods such as ivory shell.

## 5. Conclusions

This study, for the first time, integrated 16S rRNA gene sequencing and untargeted metabolomics to characterize intestinal bacterial community composition, metabolite profiles, and their associations in ivory shell reared under different water-exchange regimes. Compared with the high-water-exchange group, ivory shells in the low-water-exchange group exhibited significantly lower SGR, WGR, DISL, DISW, and intestinal T-SOD and LIP activities. Among the identified intestinal metabolic pathways, lipid-related pathways displayed the most prominent differential alterations between the two groups. Low-water-exchange conditions significantly upregulated the intestinal levels of the inflammation-related metabolites PGs and LTs, as well as the sphingolipid metabolite Sph. Correlation analyses revealed that the relative abundance of *Halodesulfovibrio* was higher under low-water-exchange conditions and correlated positively with PGs and LTs but negatively with LPC and T-SOD activity. In contrast, *Christensenellaceae_R-7_group* was less abundant under low-water-exchange conditions; its abundance was positively correlated with intestinal LIP activity and significantly associated with metabolites involved in primary bile acid biosynthesis. Overall, low-water-exchange conditions were associated with coordinated changes in growth performance, intestinal enzyme activities, microbial composition, and metabolite profiles. However, the biological mechanisms underlying these associations remain to be elucidated. These findings innovatively reveal the response characteristics of intestinal microbiota–metabolite in ivory shell under low-water-exchange conditions and provide a theoretical basis for optimizing recirculating aquaculture system (RAS) culture strategies via targeted regulation of key microbial taxa and core metabolic pathways in future research.

## Figures and Tables

**Table 1:** Growth parameters of ivory shell after 60 days of culture.

Parameters	Water Exchange Rate Group		*p* Value
	H	L	
Initial average weight (g)	2.93 ± 0.69		——
Initial mean shell length (mm)	23.32 ± 1.92		——
Initial mean shell width (mm)	15.46 ± 1.56		——
Final weight (g)	5.12 ± 1.44	4.55 ± 1.04	<0.0001
Final shell length (mm)	27.63 ± 3.33	26.67 ± 2.58	0.0060
Final shell width (mm)	18.88 ± 2.05	18.04 ± 1.78	0.0012
Specific growth rate/SGR (%/day)	1.10 ± 0.32	0.62 ± 0.22	<0.0001
Weight gain rate/WGR (%)	47.29 ± 9.75	30.47 ± 9.54	<0.0001
Daily increment in shell length/DISL (μm/d)	101.32 ± 42.99	70.96 ± 23.76	0.0060
Daily increment in shell width/DISW (μm/d)	74.11 ± 25.63	54.46 ± 17.69	0.0012

Note: Data are presented as mean ± SD of cage means (*n* = 6 cages per treatment). SD, standard deviation; H, high water-exchange group; L, low water-exchange group; SGR, specific growth rate; WGR, weight gain rate; DISL, daily increment in shell length; DISW, daily increment in shell width. A *p* value < 0.05 was considered statistically significant. *——* indicates not applicable.

**Table 2:** The α-diversity indexes of the intestinal microbiota in the H and L groups.

Group/Statistic	Shannon	Simpson	Chao	Sobs	Ace
H	4.00 ± 1.22	0.09 ± 0.08	491.12 ± 265.45	483.50 ± 264.96	488.80 ± 263.49
L	2.86 ± 0.16	0.11 ± 0.03	129.09 ± 29.85	127.67 ± 30.16	129.34 ± 30.22
*p* value	0.07	0.23	0.02	0.03	0.02
FDR-adjusted *p* value	0.08	0.23	0.06	0.06	0.06

Note: Data are presented as mean ± SD (*n* = 6 pooled intestinal biological samples per treatment). SD, standard deviation; H, high water-exchange-rate group; L, low water-exchange-rate group. Differences between groups were assessed using the Wilcoxon rank–sum test, followed by false discovery rate (FDR) correction for multiple comparisons. An FDR-adjusted *p* value < 0.05 was considered statistically significant.

**Table 3:** Differentially abundant metabolites in the pathways of KEGG enrichment analysis (VIP ≥ 1; FC ≥ 1 or FC ≤ 1; *p* < 0.05).

Pathway	Metabolite	Up/Down	*p* Value	*p* Adjust
Lipid metabolism				
Arachidonic acid metabolism			0.000001361	0.0001715
	15-Keto-Prostaglandin E2	Up		
	9S-Hydroxy-11,15-Dioxo-5Z,13E-Prostadienoic Acid	Up		
	Gpcho(20:4/14:1)	Down		
	20-Carboxy-Leukotriene B4	Up		
	Leukotriene C4	Up		
	Delta-12-Prostaglandin J2	Up		
	20-Hydroxy-Leukotriene E4	Down		
	Prostaglandin B2	Up		
	8,9-Epoxyeicosatrienoic Acid	Down		
Steroid hormone biosynthesis			0.0004731	0.01987
	Dihydrocortisol	Up		
	Cortol	Up		
	5Alpha-Dihydroprogesterone	Up		
	Estriol	Up		
	17A,21-Dihydroxy-5B-Pregnane-3,11,20-Trione	Up		
	3A,11B,21-Trihydroxy-20-Oxo-5B-Pregnan-18-Al	Up		
	Cortexolone	Up		
Primary bile acid biosynthesis			0.0004999	0.01575
	Cholic Acid	Down		
	7A-Hydroxy-Cholestene-3-One	Up		
	7A,12A-Dihydroxy-5B-Cholestan-3-One	Up		
	3Alpha,7Alpha,12Alpha-Trihydroxy-5Beta-Cholestanate	Up		
	Glycocholic Acid	Up		
Glycerophospholipid metabolism			0.008086	0.1274
	Lpc(18:3)	Down		
	Lysopc(15:0)	Down		
	Pc(22:5/0:0)	Down		
	Pe-Nme2(9M5/9D3)	Up		
	Gpcho(20:4/14:1)	Down		
	Lpc(18:0)	Down		
	Lpc(16:0)	Down		
	Lpc(20:1)	Down		
	Pe-Nme(18:1(9Z)/18:1(9Z))	Down		
	Lpc(22:4)	Down		
	Lpc(22:5)	Down		
alpha-Linolenic acid metabolism			0.02452	0.1931
	Gpcho(20:4/14:1)	Down		
	17-Hydroxylinolenic Acid	Up		
	7-Epijasmonic Acid	Up		
Global and overview maps				
Nucleotide metabolism			0.009143	0.128
	Hypoxanthine	Up		
	2′-Deoxyinosine 5′-Monophosphate	Up		
	6-Dehydrotestosterone Glucuronide	Up		
	2′-Deoxyuridine	Up		
Cancer: overview				
Choline metabolism in cancer			0.00994	0.09609
	Lpc(18:3)	Down		
	Lysopc(15:0)	Down		
	Pc(22:5/0:0)	Down		
	Gpcho(20:4/14:1)	Down		
	Lpc(18:0)	Down		
	Lpc(16:0)	Down		
	Lpc(20:1)	Down		
	Lpc(22:4)	Down		
	Lpc(22:5)	Down		
	Dg(9D5/11D5/0:0)	Down		
Small cell lung cancer			0.04239	0.2428
	Retinoic Acid	Up		
Digestive system				
Bile secretion			0.01139	0.0944
	Cholic Acid	Down		
	Pregnanediol 3-O-Glucuronide	Up		
	Uric Acid	Up		
	Leukotriene C4	Up		
	Glycocholic Acid	Up		
Immune system				
Intestinal immune network for IgA production			0.02846	0.211
	Retinoic Acid	Up		
Nervous system				
Serotonergic synapse			0.03632	0.1915
	Leukotriene C4	Up		
	Prostaglandin B2	Up		
	8,9-Epoxyeicosatrienoic Acid	Down		
Xenobiotics biodegradation and metabolism				
Drug metabolism—other enzymes			0.01327	0.1394
	Irinotecan	Down		
	Apc	Down		
	Npc	Down		

Note: H, high-water-exchange-rate group; L, low-water-exchange-rate group. “Up” and “Down” indicate higher and lower metabolite levels, respectively, in the L group relative to the H group. The “*p* Value” column reports uncorrected pathway-enrichment *p* values; a nominal *p* value < 0.05 indicates nominally significant enrichment. The “*p* Adjusted” column reports *p* values adjusted using the Benjamini–Hochberg (BH) procedure; a BH-adjusted *p* value < 0.05 indicates enrichment that remained statistically significant after correction for multiple testing.

## Data Availability

The datasets generated for this study can be found in the NCBI under BioSample accessions SAMN60265312–SAMN60265323—https://www.ncbi.nlm.nih.gov/biosample/60265312 to https://www.ncbi.nlm.nih.gov/biosample/60265323; accessed on 20 August 2026.

## Supplementary Materials

The following supporting information can be downloaded at https://www.mdpi.com/article/10.3390/biology15171473/s1. Table S1: Endpoint pH and dissolved inorganic nitrogen concentrations in rearing water under different water-exchange treatments on day 60.

## Abbreviations

The following abbreviations are used in this manuscript:

RAS	Recirculating aquaculture systems
FTS	Flow-through aquaculture systems
T-SOD	Total superoxide dismutase
LIP	Lipase
PGs	Prostaglandins
LTs	Leukotrienes
Sph	Sphingosine
Lpc	Lysophospholipids
DISL	The daily increment in shell length
DISW	Daily increment in shell width
WGR	Weight gain rate
SGR	Specific growth rate
MFIP	Modern fishery industrial parks
LC-MS	Liquid chromatography-mass spectrometry
MS	Mass spectrometry
AMS	α-amylase
TPS	Trypsin
ACP	Acid phosphatase
AKP	Alkaline phosphatase
FDR	False discovery rate
PCoA	Principal Coordinates Analysis
LEfSe	Linear Discriminant Analysis Effect Size
LDA	Linear Discriminant Analysis
UPLC-TripleTOF	Ultra-high-performance liquid chromatography coupled with time-of-flight mass spectrometry
HMDB	Human Metabolome Database
PLS-DA	Partial least squares-discriminant analysis
DAMs	Differentially abundant metabolites
BH	The Benjamini–Hochberg
RSD	Relative standard deviation
QC	Quality control
KEGG	Kyoto Encyclopedia of Genes and Genomes
VIP	Variable importance in projection
SD	Standard deviation
ANOSIM	Analysis of similarities
FC	Fold change
SRB	Sulfate-reducing bacterium
PC	Phosphatidylcholine
LDL	Low-density lipoprotein
CAT	Catalase
BAs	Bile acids
TG	Triglycerides
CA	Cholic acid
DCA	Deoxycholic acid
FXR	Farnesoid X receptor
dIMP	2′-deoxyinosine 5′-monophosphate
