# Supplementary material for: Water Exchange Rate Shapes Intestinal Microbiota–Metabolite Interactions and Physiological Responses in Babylonia areolata
Source: Biology (Basel). 2026 Sep 1;15(17):1473. doi: 10.3390/biology15171473 (PMC13565011; doi:10.3390/biology15171473)
Supplement: Supplementary file 1 [file biology-15-01473-s001.zip › biology-4477680-supplementary.pdf]

Supplementary Material

Supplementary Table S1. Endpoint pH and dissolved inorganic nitrogen concentrations in rearing water under different water-exchange treatments on day 60.

Water exchange rate group	pH	NO_3^--N (mg·L^{-1})	NO_2^--N (mg·L^{-1})	NH_4^+-N (mg·L^{-1})
H	8.06±0.07	1.56 ± 0.69	0.016 ± 0.004	0.047 ± 0.03
L	7.87±0.05	14.86 ± 1.40	0.011 ± 0.002	0.027 ± 0.02

Note: Data are presented as mean ± SD (n = 6). SD, standard deviation; H, high water-exchange group; L, low water-exchange group; NO_3^--N, nitrate nitrogen; NO_2^--N, nitrite nitrogen; NH_4^+-N, ammonia nitrogen.
